# Effect of biased feedback on motor imagery learning in BCI-teleoperation system

**DOI:** 10.3389/fnsys.2014.00052

**Published:** 2014-04-09

**Authors:** Maryam Alimardani, Shuichi Nishio, Hiroshi Ishiguro

**Affiliations:** ^1^Department of System Innovation, Graduate school of Engineering science, Osaka UniversityOsaka, Japan; ^2^Advanced Telecommunications Research Institute InternationalKyoto, Japan

**Keywords:** body ownership illusion, BCI-teleoperation, motor imagery learning, feedback effect, training

## Abstract

Feedback design is an important issue in motor imagery BCI systems. Regardless, to date it has not been reported how feedback presentation can optimize co-adaptation between a human brain and such systems. This paper assesses the effect of realistic visual feedback on users' BCI performance and motor imagery skills. We previously developed a tele-operation system for a pair of humanlike robotic hands and showed that BCI control of such hands along with first-person perspective visual feedback of movements can arouse a sense of embodiment in the operators. In the first stage of this study, we found that the intensity of this ownership illusion was associated with feedback presentation and subjects' performance during BCI motion control. In the second stage, we probed the effect of positive and negative feedback bias on subjects' BCI performance and motor imagery skills. Although the subject specific classifier, which was set up at the beginning of experiment, detected no significant change in the subjects' online performance, evaluation of brain activity patterns revealed that subjects' self-regulation of motor imagery features improved due to a positive bias of feedback and a possible occurrence of ownership illusion. Our findings suggest that in general training protocols for BCIs, manipulation of feedback can play an important role in the optimization of subjects' motor imagery skills.

## Introduction

Brain computer interfaces (BCIs) have widely become popular in many fields as a new communication and control channel between the human brain and an external device. However, the application of this technology is not as simple and intuitive as its concept suggests. To operate a BCI, subjects need to perform certain tasks and learn how to intentionally modulate certain characteristics of their brain activities in order to express their intentions. The motor imagery method, for instance, is one of the most commonly employed methods for BCI control of intended motions (Curran and Stokes, 2003). Subjects imagine the movement of a certain limb of their own body to induce changes in mu and beta rhythms over the corresponding sub-region of sensorimotor cortex. These changes are detected by BCI and translated into control commands. Motor imagery task requires relatively longer training compared to other BCI paradigms such as P300 or steady state visually evoked potential (SSVEP) since the mental rehearsal of a movement without actual execution is not a normal and daily practice for subjects and hence the task of motor imagery is an unfamiliar experience to most of them.

While the importance of subject's motor imagery skills in BCIs is well recognized, most studies have focused on the computer side and improving classification algorithms and very few have attended the human side and training paradigms that can facilitate the skill acquisition process for subjects (Lotte et al., 2013). As in any form of interface, users of BCIs learn to co-adapt with the system through the feedback they receive of their performance. Therefore feedback design is particularly influential in the process of motor imagery learning and performance improvement. Standard BCI protocols typically provide online visual feedback in the form of a moving cursor or target on the computer screen. Neuper et al. compared realistic presentation of feedback, in form of a grasping hand vs. abstract feedback in the form of an extending bar, on a computer screen (Neuper et al., 2009). However, they found no evidence of a significant difference between the performances of two feedback groups. In another study, the influence of motivation on BCI performance was also investigated by biasing the feedback accuracy (Barbero and Grosse-Wentrup, 2010). The results indicated that subjects with poor performance benefitted from positive biasing while those with better performance were impeded by inaccurate feedback. In a similar work (Gonzalez-Franco et al., 2011), authors provided subjects fake negative and positive feedback of their performance and reported that negative feedback had a greater learning effect on motor imagery BMIs.

Although in the above works, the effect of feedback presentation and accuracy has been probed, none of them has actually discussed the direct interaction between subject and BCI system. When performing a motor imagery task, subjects are asked to imagine their own body movements while the output is fed back in the form of movement for objects other than their own body. This mismatch and dissociation between subject's life experience and BCI task can in fact interfere with the imagination and impair the performance of motor imagery especially for novice users.

The goal of the present study is to explore the influence of feedback design on enhancement of user's performance and interaction with a BCI system. Previously we showed that BCI-operation of a pair of humanlike robotic hands by motor imagery while watching first-person perspective images of robot's movement could induce an illusion of body ownership transfer (BOT) for the operators (Alimardani et al., 2013). In post-experiment interviews, some subjects stated that when the robot moved as they intended, it felt like their own hand was moving and motor imagery became easier. We hypothesize that inducement of such feeling of ownership and the sense of agency driven toward the seen motions may have a positive loop effect on execution of motor imagery during BCI-operation. In other words, we speculate that once the thought of “I am the one moving the hands” raises the feeling of “These hands are mine,” the illusion of owning hands enhances the imagery ability in subjects and boosts the inverse thought of “These are my hands so I can move them.”

To that end, in this study we used the same BCI-teleoperation paradigm while exposing naïve subjects to different feedback conditions in order to probe the relationship between subject's experience of BOT and BCI-performance. Two experiments are presented. In the first experiment, by manipulating the presentation of misperformance, we surveyed how subjects' perception of their own performance could affect the intensity of BOT. In the second experiment, we then examined how this effect can be influential on subjects' real performance and trend of motor imagery learning.

Both experiments in this study were approved by the Ethics Committee of Advanced Telecommunications Research Institute International (12-506-3). All subjects read and signed a written consent form prior to experiment and received payment for their participation.

## Experiment 1

This experiment was designed to investigate the inducement of body ownership illusion for a pair of BCI-operated human-like robotic hands under different presentations of feedback.

### Participants

Forty healthy participants (26 male, 14 female, age *M* = 21.13, *SD* = 1.92) were selected for the experiment. Thirty eight participants were right-handed and 2 left-handed. All participants were naive to the research topic and received explanation prior to the experiment.

### Method

Participants sat in a comfortable chair and were asked to remain motionless. They wore an EEG electrode cap and 27 EEG electrodes were placed over their primary sensori-motor cortex according to the international 10–20 system (FT7, FC5, FC3, FC1, FCz, FC2, FC4, FC6, FT8, T7, C5, C3, C1, Cz, C2, C4, C6, T8, TP7, CP5, CP3, CP1, CPz, CP2, CP4, CP6, TP8). A reference electrode was mounted on the right ear and a ground electrode on the forehead. Participants were asked to imagine a grasp or squeeze motion for their own hand while their cerebral activities were recorded by g.USBamp biosignal amplifiers (Guger Technologies). In an initial training session, they practiced a motor imagery task by extending a feedback bar to left or right side on a 15-inch laptop computer screen. A visual cue in the form of a horizontal pointing arrow specified the timing and the hand they were supposed to hold image for. Each trial lasted 7.5 s and started with the presentation of a fixation cross on the display. After 2 s an acoustic warning was given in the form of a “beep.” From second 3 to 4.25, an arrow pointing to the left or right side randomly was shown. Depending on the arrow's direction participants were instructed to perform motor imagery. They watched the feedback bar and continued the imagery task until the fixation cross was erased. After a short pause, which took 1 second, the next trial started. The first run consisting of 40 trials (20 trials per class left/right presented in a randomized order) was conducted without feedback and lasted 5 min. The recorded brain activities in the initial non-feedback run were used to set up a subject specific classifier for the classification in the following feedback runs. In the feedback runs, participants performed similar trials but received online classification results of their performance in form of a horizontal feedback bar on the screen. Subjects' task was to extend the feedback bar in the correct direction.

The classification of recorded signals was conducted under Simulink/MATLAB (Mathworks) for offline and online parameter extraction. This process included bandpass filtering between 0.5 and 30 Hz, sampling at 128 Hz, cutting off artifacts by notch filter at 60 Hz, and adopting the Common Spatial Pattern (CSP) algorithm for discrimination of Event Related Desynchronization (ERD) and Event Related Synchronization (ERS) patterns associated with the motor imagery task (Guger et al., 2000). The classifier was trained using CSP analysis of calibration measurements. CSP found weight vectors that weighed each electrode based on its importance for the discrimination task. The spatial filters were designed such that the resulting signal had maximum variance for left trials and minimum variance for right trials. Therefore, the difference between left and right populations was maximized to show where the EEG variance fluctuated the most. Finally, when the discrimination between left and right imaginations was made, the classifier outputted a linear array signal in the range of [−1,1], where −1 denotes the extreme left and 1 denotes the extreme right. Negative values were then translated as the robot's left hand grasp motions and positive values as the robot's right hand grasp motions. A threshold of ±0.1 was considered in the system, in order to avoid multiple movements of both hands for subjects with unstable classification results.

**Figure 1:**
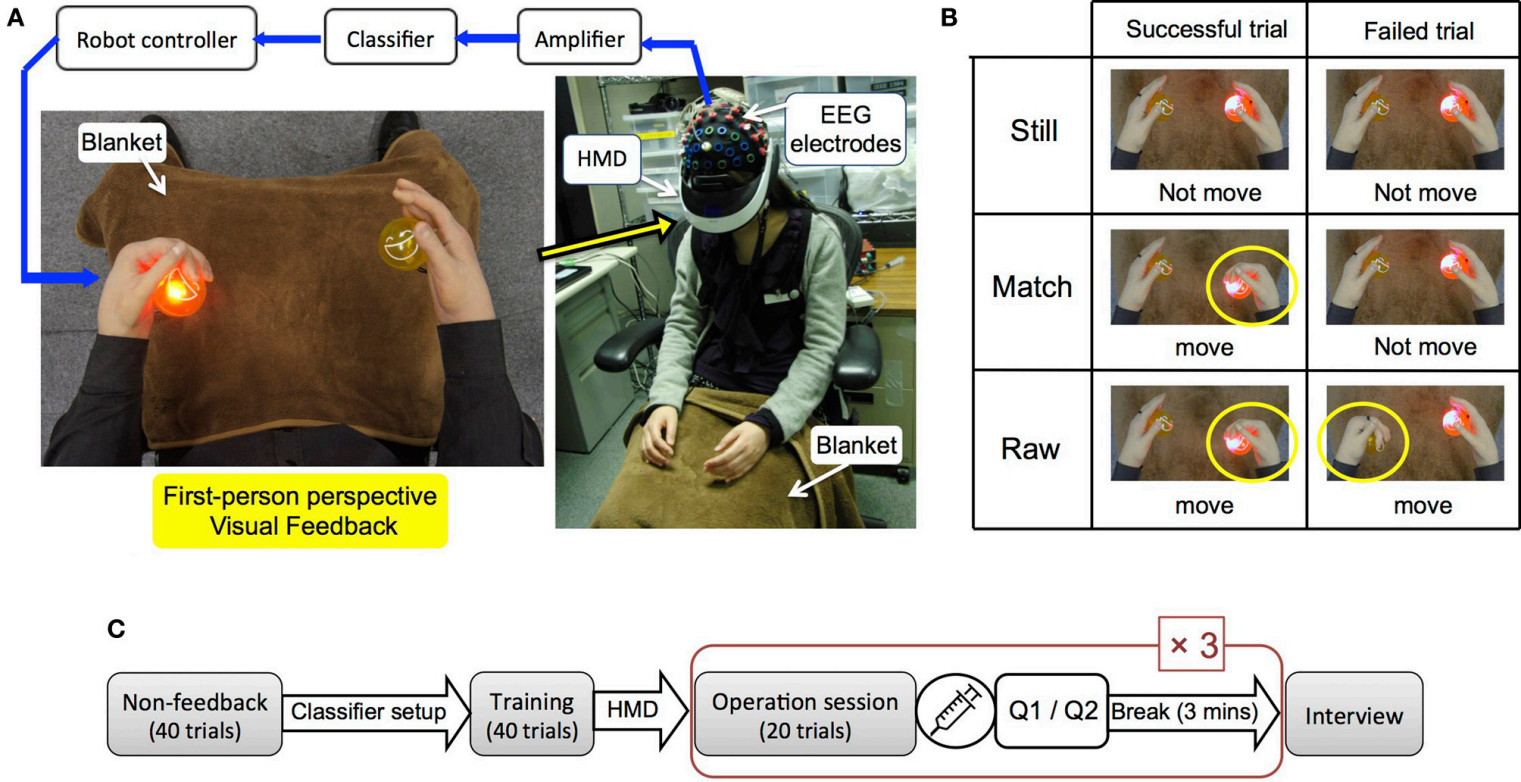
**Experiment setup. (A)** EEG electrodes installed on a subject's sensorimotor cortex recorded brain activities during a motor imagery task. Subjects watched first-person images from a robot's perspective through a head mounted display. A lighting ball in front of each of the robot's hands gave motor imagery cues and subjects imagined grasping their corresponding hands. The classifier detected two classes of results (right or left) and motion command was sent to the robot's hand. **(B)** Subjects repeated the experiment under three different conditions: Still, where the robot's hand didn't move at all. Match in which the robot's hand only moved in successful trials, and Raw where the robot's hand also performed failed trials using the wrong hand. **(C)** Experimental procedure consisted of a non-feedback session for classifier setup, training sessions and three operational sessions.

Following training sessions the main test sessions commenced, in which subjects wore a head mounted display (Vuzix iWear VR920) and tele-operated the robot's hands using the same BCI system. They performed a motor imagery task for their right or left hand while they watched first-person images of the robot's hands performing the motions respectively (Figure 1A). Two LED-embedded balls were installed in the robot's grasp and provided motor imagery cues by randomly lighting. During the experiment subjects were told to look down as if they were watching their own hands and the same blankets were laid on both the robot's and participants' legs to give a similar view. Participants placed their arms in a similar position and orientation as the robot's arms. In order to measure subjects' physiological reactions to a threatening stimulus, skin conductance response (SCR) electrodes were installed on their left palms. A bio-amplifier recording device (Polymate II AP216, TEAC, Japan) with sampling rate of 1000 Hz was used to record SCR measurements. Prior to the testing sessions, participants watched an act of injection via a syringe to the robot's hand (painful stimulus) through the head mounted display, which was explained to them as a necessary procedure for preparing the robot. The injection was continued until subjects' SCR responses disappeared (Armel and Ramachandran, 2003). Afterwards, testing sessions were carried out in a random order under three conditions (Figure 1B):

1. Still (no feedback): The robot's hands did not move at all throughout the whole session although a subject performed motor imagery according to cues.
2. Match (no negative feedback): The robot's hands moved only in those trials that the classification result was correct and in accordance with cue.
3. Raw: The robot's hands moved according to the classification results in all trials. In case of wrong result that was not in accordance with cue, the robot's opposite hand moved.

Still was designed as control condition where visual images of hands without motion feedback were expected to raise no body ownership illusion. The last session is called Raw since we inputted the unprocessed values obtained from the classifier as the robot's motion parameter. In all conditions above, participants performed trials that were designed to be identical to the trials in training sessions regarding duration and stimulus timing. Each session was followed by a break of 3 min. Test sessions comprised 20 trials, lasting 2 min and 40 s each (Figure 1C). Following the last trial, an injection was applied to the robot's left hand to examine if the illusion of ownership could cause a response to a pain-causing stimulus (Nishio et al., 2012). Immediately after injecting the session was terminated and participants were orally asked the following questions: (Q1) When the robot's hand was injected, did it feel as if your own hand was receiving the injection? (Q2) Throughout the entire session while you were operating the robot's hands, did it feel as if they were your own hands? Participants scored Q1 and Q2 based on the seven-point Likert Scale, 1 meaning, “Didn't feel such thing at all” and 7 meaning, “Felt it very strongly.” In addition to the self-assessment, we physiologically measured the body ownership illusion by recording the SCRs.

### Result

**Figure 2:**
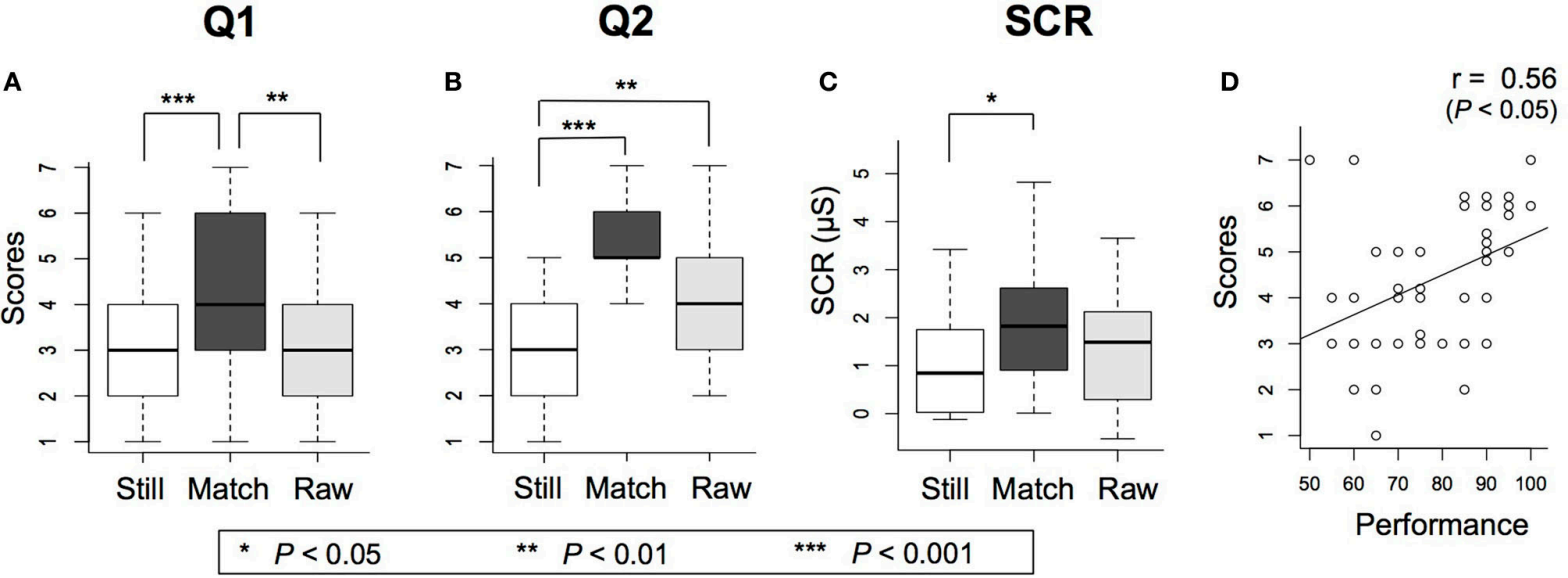
**Results for experiment 1 (A)** Mean values and standard deviations for Q1 in each session, with Match significantly higher than Still and Raw **(B)** Mean values and standard deviations for Q2 in each session, with Match and Raw significantly higher than Still **(C)** Mean values and standard deviations of SCR values for 35 subjects, with Match associated with significantly higher responses to injection than Still **(D)** Subjects' performances vs. their scores of illusion in the Match condition. For those with the same performance and score, the score has been slightly modified to a non-integer neighbor value to avoid the overlap of the markers. A significantly positive correlation was found between BCI-performance and intensity of illusion.

The response variables for 40 participants were obtained from questionnaires and SCR recordings. Participants' responses in three conditions of Still, Match and Raw were averaged and compared. The mean value, standard deviation, and *p*-value are depicted on each graph (Figure 2).

For both Q1 and Q2, the Match condition showed a higher average value compared to the other two conditions (Figures 2A,B). Non-parametric statistical analysis was used to compare as Shapiro-Wilks test rejected the normal distribution for the Likert scores. Kruskal-Wallis tests showed a significant effect of BCI tele-operation on the level of BOT between the three Still, Match and Raw conditions for Q1 scores [χ^2^_(2)_ = 19.11, *p* < 0.0001] and for Q2 scores [χ^2^_(2)_ = 34.52, *p* < 10^−6^]. *Post-hoc* Mann-Whitney *U*-tests for comparison with Bonferroni adjustment indicated that in Q1 Match (*M* = 4.38, *SD* = 1.51) raised BOT significantly higher than Still (*M* = 2.83, *SD* = 1.43); [Match > Still, *p* < 0.0001] and than Raw (*M* = 3.15, *SD* = 1.57); [Match > Raw, *p* < 0.01]. Similarly, Mann-Whitney *U*-tests with Bonferroni adjustment for Q2 scores showed significant difference between Match (*M* = 5.15, *SD* = 1.10) and Still (*M* = 2.93, *SD* = 1.25); [Match > Still, *p* < 10^−6^] and also between Raw (*M* = 4.18, *SD* = 1.38) and Still; [Raw > Still, *p* < 0.01].

The SCR peak value within a 6-s interval (1 s after the appearance of syringe in the participant's view to 5 s after the injection) was selected as the reaction value (Alimardani et al., 2013). In this experiment, we only evaluated the response values of 35 participants, since five participants showed unchanged responses during the experiment and were excluded from analysis. Results showed a higher mean value for the Match condition compared to the other two conditions (Figure 2C). Due to non-normal distribution of SCR values revealed by Shapiro-Wilks test, we performed Kruskal-Wallis tests on participants' reaction values and the analysis was significant, [χ^2^_(2)_ = 8.39, *p* < 0.01]. *Post-hoc* comparisons by Mann-Whitney *U*-tests with Bonferroni adjustment confirmed significant differences only between Match (*M* = 1.68, *SD* = 1.98) and Still (*M* = 0.90, *SD* = 1.42); [Match > Still, *p* < 0.05]. Moreover, a significantly positive correlation was found between subjects' performances and the Q1 scores only in the Match condition (Pearson correlation coefficient *r* = 0.56, *p* < 0.05, Figure 2D). The term performance here refers to the rate of trials subjects could successfully grasp the lightened ball out of the total 20 trials in each run scaled in percentage.

### Discussion

In study 1, we investigated the inducement of body ownership illusion for a pair of BCI-operated human-like robotic hands under different feedback conditions. Results from both measurement methods (Q1 and SCR) indicated significantly high responses to the injection in the Match condition, where robot's hands moved only if the classification result was correct and same as the cue. This shows that the feeling of receiving an injection was significantly stronger when the robot's hands moved exclusively in agreement with the operator's intentions than when the robot made no motion (Still) or performed a wrong motion in the case of errors (Raw). Since this is a feeling aroused due to the illusion of ownership over robot's body, we can state that the transfer of body ownership could be evoked more reliably by precise mind-control of a robot's hands.

On the other hand, in Q2 participants directly scored their sensation of ownership for robot's body during the entire operation time. Based on participants' assessments, the feeling of ownership was significantly stronger in both the Match and Raw conditions, when the robot dynamically moved and reacted to the participant's intentions, compared to the control condition, Still, when the robot did not show any motion at all. Although Match showed a higher average response compared to Raw, no significant difference between these two conditions was confirmed in Q2. This can imply that in both the Raw and Match conditions the robot's successive motions following the participant's act of motor imagery raised a sense of agency during the session that led to a perception of owning the hands in participants.

Meanwhile, the results of this experiment showed a wide dispersion over the response values of illusion in each condition, which we presumed is due to the difference of performance level among subjects. A positive correlation was confirmed between participants' performances and their indicated scores for Q1 (Figure 2D), which indicates that participants with a better operational performance experienced a stronger illusion of BOT. Therefore, we can tell that subjects' skill in a motor imagery task and BCI performance are associated with the intensity of ownership illusion in such a tele-operational system.

**Figure 3:**
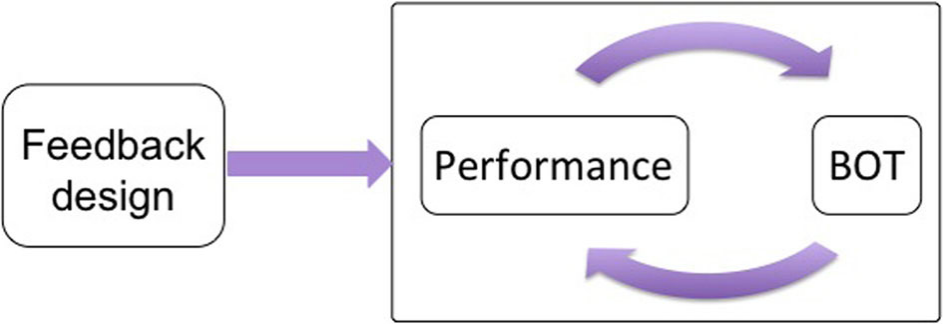
**Model diagram for effect of feedback design**. Feedback bias can affect the interaction between BCI-performance and BOT illusion.

From the obtained results in this experiment, we conclude that in a BCI-teleoperation system for humanlike hands, the feedback presentation could affect the eliciting of ownership illusion over the controlled hands; the illusion was augmented when negative feedback of subject's misperformance was eliminated. Also, a positive correlation was found between the intensity of BOT and subjects' performance, which suggests that subjects with better BCI-performance experienced a stronger illusion. Therefore BOT could be affected both by subjects' BCI-performance, and feedback design, which regulated subjects' perceptions of their own performance. On the other hand, although an intuitive conclusion of this experiment could be that better BCI-performance caused a stronger perception of illusion in subjects, the reverse thought could also be claimed; that is, higher BOT motivated subjects to perform better on the motor imagery task. Therefore, it remains to be clarified how the mutual interaction between performance and BOT is formed and how feedback design contributes to improvement of each element and their interaction (Figure 3). Thus, in experiment 2 we focused on the effect of feedback design on a subject's BCI-performance and examined how manipulation of subjects' perceptions of self-performance can affect the trend of their motor imagery learning and BCI-performance.

## Experiment 2

From experiment 1 we found that subjects' perceptions of their own performance was important for the inducement of ownership illusion. Moreover, a close relationship between the intensity of illusion and a subject's performance was found. We were further interested in knowing how the subjects' self-evaluations and subsequent inducement of BOT can directly affect their skills in a motor imagery operational system. Therefore in this experiment, by manipulating the presentation and accuracy of subjects' performances we designed four different feedback conditions including two in which each subjects' performance was positively and negatively biased in the first half of each session. We then examined how conditioning feedback can affect the trend of learning by two methods; (1) measuring subjects' online performance in the second half of sessions and (2) comparing time-variant distribution of EEG features regarding right and left hand imagery in each half of the session.

### Participants

Sixteen healthy subjects (6 male and 10 female, age *M* = 21.1, *SD* = 1.4) participated in this experiment. 15 participants were right-handed and one left-handed. None of them had participated in our previous experiments and they were all unfamiliar with the research topic. Participants received explanation prior to the experiment.

### Method

The BCI devices, preparation procedure and session paradigms of Experiment 2 were identical with Experiment 1, except in this experiment we used a new type of head mounted display (Sony HMZ-T1) for the real time first-person visual feedback (Figure 1A).

Participants performed 4 experimental sessions each consisting of 40 imagery trials which lasted 5 min. Each session was followed by a break of 3 min. The first half of each session (20 trials) was randomly conditioned as below:

1. Raw: Participants' performance was not biased. The robot's hands grasped the ball according to the classification result.
2. Match: Participants' performance was not biased. However, the robot's hands only grasped the lit ball when the classification results matched the cue.
3. Positive Feedback (Fake-P): Participants' performance was biased positively. The robot's hands grasped the lit ball correctly in 90% of trials regardless of the subject's real performance.
4. Negative Feedback (Fake-N): Participants' performance was biased negatively. The robot's hands grasped the lit ball correctly only in 20% of trials regardless of the subject's real performance.

In the first two conditions, Raw and Match (Figure 1B), a subject's performance was not biased although the robot's hand motion in mistaken trials differed—one with execution of wrong hand motion and one without robot motion. Raw is an equivalent feedback design of the one used in general motor imagery BCIs where subject's failure in executing motor imagery for one class results in feedbacks of the other class. However, since Raw and Match conditions previously revealed a different level of illusion (Experiment 1) we made the assumption that presentation of negative feedback affects subject's perception of self-performance and therefore influences subject's interaction with the system. To clarify this point, we respectively designed two more sessions (Fake-P and Fake-N) in which we deliberately biased feedback of performance regardless of subjects' real performance accuracy in order to extremely enhance or decrease their self-evaluation. In the second half of all sessions subjects received feedback of their real performance as they did in Raw. The goal was to seek changes in BCI-performance and motor imagery skills in the second half of each session due to the positive or negative bias of feedback. Subjects' performance in the second half of all sessions was registered.

In addition to subjects' online performance, we conducted an offline re-analysis of data to extract the feature distribution of right and left motor imagery in each session. We speculated that by receiving biased feedback or experiencing illusion, subjects may consciously or unconsciously modify the generation of their brain activity patterns throughout the experiment, although the classifier could fail to detect these changes because it did not use a learning algorithm and once the classification boundary for the two classes, Right and Left, was defined within the feature space in the initial training session, the same classifier and parameters were used to the end of the experiment. Therefore, we used the original brain signals and ran the following offline processing to seek changes in motor imagery patterns.

After artifact removal and temporal filtering (Guger et al., 2000), the features used for classification were obtained by the method of CSP. Having N channels of EEG for each left and right trial ***X***, the CSP builds an *N* × *N* projection matrix ***W***. With the projection matrix ***W***, the mapping of a trial is given as

Z=WX

The columns of ***W*^−1^** are the CSPs and can be seen as time-invariant EEG source distribution vectors. By design the variance for imagining left hand motion is largest in the first row of ***Z*** and decreases with the increasing number of subsequent rows. To obtain reliable features, it is not necessary to calculate the variances of all *N* time series. The optimal number of CSPs used to build the feature vector is four (Müller-Gerking et al., 1999). Therefore, only the first and last two rows (*p* = 4) of ***W*** were used to filter data ***X*** and build a new signal ***Z_p_*** (*p* = 1… 4). The variance of the resulting four time series is obtained for a time window *T* = (*t*_0_, *t*_1_)

$$var_{p}=\sum_{t=t_{0}}^{t_{1}}{\left(Z_{p(t)}\right)}^{2}$$

where window length was set to be 1 s, starting 1500 ms after the presentation of the cue (Pfurtscheller and Neuper, 2001). Feature vectors were obtained after normalizing and log-transforming as following:

$$f_{p}=log\left(\frac{var\left(Z_{p}\right)}{\sum_{i=1}^{p}var\left(Z_{p}\right)}\right)$$

The online classifier uses each trial's feature vector ***f_p_*** to categorize it into two classes of right and left. In order to estimate the goodness of this classification, we used Fisher's discriminant criterion measures in a linear discriminant analysis to observe the distribution of two classes feature vectors in a 4-dimential space. Fisher's parameter ***J*** is defined as

$$J=\frac{{\left|{\tilde{\mu }}_{R}-{\tilde{\mu }}_{L}\right|}^{2}}{{\tilde{s}}_{R}^{2}+\;{\tilde{s}}_{L}^{2}}$$

where ${\tilde{\mu }}_{R}$ and ${\tilde{\mu }}_{L}$ are the means of feature vectors for two right and left classes and the quantity ${\left|{\tilde{\mu }}_{R}-{\tilde{\mu }}_{L}\right|}^{2}$ is the distance between the two classes' means. For each class ${\tilde{s}}_{R}^{2}$ and ${\tilde{s}}_{L}^{2}$ were defined as the scatter, an equivalent of the variance, and obtained by

$${\tilde{s}}_{i}^{2}=\sum_{x\in f_{i}}{\left(x-\;{\tilde{\mu }}_{i}\right)}^{2}$$

The quantity ${\tilde{s}}_{R}^{2}+\;{\tilde{s}}_{L}^{2}$ indicates the within-class scatter. When performing motor imagery a larger ***J*** corresponds to closer dispersion of feature vectors per each class and further distance between two class means, which represents better feature distribution for classification, and therefore better execution of motor imagery task.

In each session, the ***J*** parameter for the first 20 conditioned trials (***J*_1_**) and for the second 20 test trials (***J*_2_**) was calculated. Since subjects' initial skills were diverse, and for every subject the order of sessions was considerable in the amount of motor imagery skills, the ratio **Δ*J* = *J*_2_**/***J*_1_** was selected as a measurement of subjects' motor imagery learning in that session.

### Result

#### Online performance

**Figure 4:**
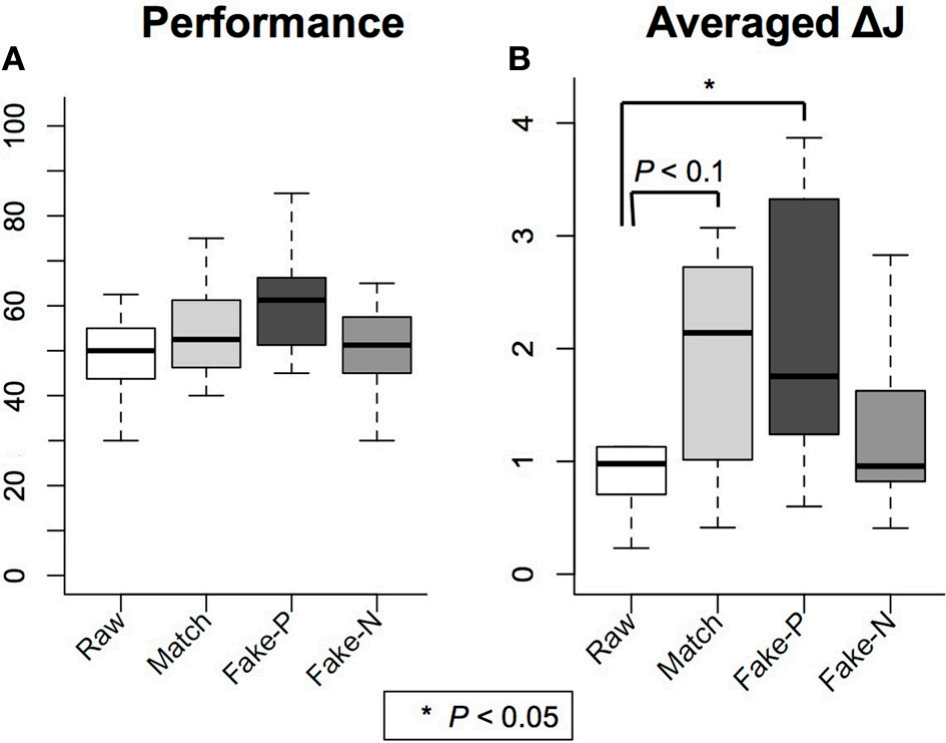
**Results for experiment 2 (A)** Mean value of subjects' performances in the second half of each session is demonstrated. No significant difference was found. **(B)** Mean value of the ratio ***J*_2_**/***J*_1_**, an identifier of motor imagery quality, showed significantly higher values in the Fake-P and Match conditions compared to Raw.

Performances of 16 subjects in the second half of each session were averaged and demonstrated in Figure 4A. The term performance refers to the percentage of successful trials among the post 20 trials. Fake-P (*M* = 60.78, *SD* = 10.24) showed the highest performance compared to Raw (*M* = 49.22, *SD* = 9.07), Match (*M* = 54.37, *SD* = 10.89) and Fake-P (*M* = 50.47, *SD* = 10.58). However, One-Way ANOVA test did not reject the null hypothesis, [*F*_(3, 60)_ = 2.51, *p* = 0.07].

#### Offline classification

We calculated **Δ*J*** for 16 subjects. Using interquartile range (IQR) for statistical dispersion in each condition (Moore and McCabe, 1998), two outliers were detected in the Fake-N condition (S2 and S4) and one outlier was detected in the Raw condition (S15). The data of these three subjects were discarded from further analysis. The mean value of **Δ*J*** for the remaining 13 subjects was highest in the Fake-P condition (*M* = 2.12, *SD* = 1.78) compared to the other three conditions, Raw (*M* = 1.07, *SD* = 0.75), Match (*M* = 1.87, *SD* = 0.95) and Fake-N (*M* = 1.35, *SD* = 0.84) (Figure 4B). A One-Way ANOVA test yielded a significant effect of feedback design for the four conditions, [*F*_(3, 48)_ = 3.53, *p* < 0.05]. Using Tukey *post-hoc* comparisons, a significance difference was obtained between Fake-P and Raw; [Fake-P > Raw, *p* < 0.05] and between Match and Raw; [Fake-P > Raw, *p* = 0.08].

### Discussion

In experiment 2, we biased the visual feedback of performance in a BCI-teleoperation system of a human-like robot in order to probe the effect of positive and negative feedback on subjects' BCI-performance and motor imagery skills.

Online results demonstrated no significant changes in the subjects' real time performances and the mean value of subjects' performances remained in the chance level for all conditions. On the other hand, results from offline classification revealed that the ratio ***J*_2_**/***J*_1_**, an identifier of class separation between the two halves of sessions, was significantly higher in the Fake-P than in the Raw condition. This indicates that subjects could generate motor patterns that are more classifiable by CSP algorithm by receiving positive feedback of their performance in the Fake-P condition. Using a statistical significance level of 10%, a similar relation was confirmed between Match and Raw conditions, indicating that in the Match condition where subjects did not receive negative feedback of their failed performance, motor imagery improved and they could produce more separable activity patterns for two classes of right and left hand movement. Both results imply that positive bias of feedback had an enhancing effect on motor imagery learning which is consistent with some previous reports (Lotte et al., 2013). One probable cause could be the inducement of a stronger BOT due to biased feedback, which facilitated imagination of movement in motor imagery task and eventually enhanced self-regulation of brain patterns in subjects (Figure 3).

Unlike previous reports on biased BCI feedback, no significant improvement (Gonzalez-Franco et al., 2011) or impediment (Barbero and Grosse-Wentrup, 2010) was found in the Fake-N condition compared to other conditions. However, S2 and S4 who were discarded from analysis as outliers showed drastic **Δ*J*** increase in Fake-N. Since subjects majorly received enhanced learning in Fake-P condition, we assume that the effect of biasing is closely relevant to the subject's personality and the influence of motivation on different individuals. While there are learners who benefit from encouragement and positive feedback of their performance, there are a few who benefit more from negative feedback and try harder when the feedback informs them that they are not performing well. In future experiments, a personality test could be used in order to categorize subjects into groups, so that results can be analyzed according to stratified personality groups.

Lastly, although in this experiment we hypothetically assume that enhancement of motor imagery learning due to positive bias of feedback was associated with ownership illusion over the controlled robot's hands (Figure 3), further study is required to veritably measure the intensity of illusion at the end of each conditioned section. In this experiment we suspected that pausing the sessions and asking assessment questions could shatter the illusion. In the future, comparison between human-like and non-human-like visual feedback under biased feedback is necessary to precisely verify whether illusion of body ownership influences the trend of motor imagery learning.

## Conclusion

In this study, we designed two experiments to answer the following questions: (1) How can presentation of visual feedback affect the inducement of body ownership illusion in the BCI-operators of human-like hands, and (2) How can positively and negatively biased feedback in such a system influence operators' interaction with the system and improve their BCI performances. Results of the first experiment revealed that negative feedback of subjects' errors impeded the intensity of ownership illusion. Also BOT was correlated with subjects' performance in BCI and how well subjects felt they were in control of the hands. In the second experiment, we realized that biasing feedback could not immediately boost subjects' performance in the same session. However, the analysis of brain patterns showed that in fact it could change the trend of motor imagery learning.

In terms of feedback design for future BCI systems, it is conceivable that a more realistic feedback presentation can assist novice users to train and adapt to a system faster and more efficiently. Also, BCI users may benefit from positive bias of feedback in training sessions, although their personality should be taken into account. Meanwhile, since subjects motor imagery skills dynamically change during a session based on their state of mind, further development of sophisticated classifiers that customize classification parameters in an online session are required.

### Conflict of interest statement

The authors declare that the research was conducted in the absence of any commercial or financial relationships that could be construed as a potential conflict of interest.
